# Menstrual Blood as a Non-Invasive Alternative for Monitoring Vitamin Levels

**DOI:** 10.3390/jcm13237212

**Published:** 2024-11-27

**Authors:** Amy L. Whitbread, Lucas Mittelmeier, Rajnish P. Rao, Wolfram Mittelmeier, Katrin Osmanski-Zenk

**Affiliations:** 1 The smart period blood GmbH, D-10119 Berlin, Germany; amy@theblood.io (A.L.W.); rajnish@theblood.io (R.P.R.); 2 Orthopedic Clinic and Policlinic, Rostock University Medical Center, D-18057 Rostock, Germany; lucas.mittelmeier@googlemail.com (L.M.); wolfram.mittelmeier@med.uni-rostock.de (W.M.)

**Keywords:** menstrual blood, menstruation, vitamin A, vitamin D, dried blood spots, capillary blood

## Abstract

**Background/Objectives**: Menstrual blood has recently emerged as a novel specimen for diagnostics, offering a non-invasive alternative to traditional blood testing methods. Despite the importance of vitamins and monitoring their levels in preventative healthcare measures, the feasibility of measuring them in menstrual blood has yet to be explored. In this study, we aimed to assess the potential of using menstrual blood for determining vitamin levels by comparing their levels in menstrual blood to those in matched capillary blood samples. **Methods**: A prospective, monocentric, observational study was conducted with healthy, reproductive-aged voluntary participants. Menstrual blood was collected from 30 participants using a menstrual cup, and the corresponding capillary blood samples were obtained using a finger prick. The samples were transferred to dried blood spot (DBS) cards and analyzed using mass spectrometry to determine vitamin levels. Statistical analyses were performed to compare menstrual blood vitamin A and D levels, and hemoglobin, to those in capillary blood. **Results**: The vitamin levels could be ascertained from the menstrual blood, and were observed to significantly correlate with those from the capillary blood for both vitamin A (*r* = 0.77, *p* < 0.001) and vitamin D (*r* = 0.66, *p* < 0.001), despite being statistically different. **Conclusions**: The results of this pilot study demonstrate the potential utility of menstrual blood in estimating vitamin A and D levels, illustrating the prospect of a non-invasive menstrual blood-based vitamin test following larger clinical and analytical validation studies.

## 1. Introduction

Vitamins, essential micronutrients required in minute quantities, are pivotal for numerous cellular functions [1]. Despite their importance, the human body lacks the ability to synthesize them in adequate amounts, necessitating additional intake through diet or supplementation [1]. Due to the key roles vitamins play in many physiological functions [2], including physical [3], cognitive [4,5], and reproductive health [6,7,8], maintaining optimal concentrations is crucial for overall health and well-being [9]. Certain vitamins also play multifaceted roles contributing to all of these physiological functions, as is the case for vitamin D [10]. Vitamin D is known for its importance in physical health with being crucial for maintaining bone and muscle function [11], but increasing studies are also uncovering its potential roles in cognitive [12,13] and reproductive health [14,15]. The role of vitamin D in reproductive health is seemingly a significant one, influencing both male and female fertility, although the exact mechanisms remain to be fully elucidated. For females, vitamin D has been shown to influence various reproductive hormones [16], with deficiencies associated with menstrual cycle irregularities [17] and reproductive disorders such as polycystic ovarian syndrome (PCOS) [18], and has implications for fertility outcomes [19]. Monitoring vitamin levels, such as vitamin D, is, therefore, particularly important for permitting the early detection of deficiencies, allowing for proper management, and potentially preventing escalation to more significant health concerns. The ability to measure specific vitamin levels is consequently considered a cornerstone of preventative healthcare and personalized medicine [20].

The traditional methods of measuring vitamin levels typically involve obtaining blood samples through venipuncture or capillary blood sampling. Venipuncture, long considered the gold-standard method for diagnostic testing in clinical medicine [21], is favored due to the large volume of blood acquired and the extensive array of diagnostic tests available. However, as this method is invasive, it can be performed only by a trained professional in a clinical facility and is, thus, both time- and resource-consuming [22].

Capillary blood sampling, on the other hand, offers a more convenient and less invasive alternative [23]. This method, which involves obtaining a small volume of whole blood through a puncture on the finger, has revolutionized the approach to blood sampling. The simplicity and minimal invasiveness of capillary blood sampling make it an ideal method for at-home collection, thereby promoting a patient-centered approach to healthcare. By enabling individuals to collect their samples at their own convenience, it reduces the need for clinical visits [24]. Advancements in clinical chemistry and the evolution of dried blood spot (DBS) technology have complemented this, with dried capillary blood on blotting paper offering prolonged sample stability and simpler transportation [25,26]. Therefore, capillary blood sampling presents an advantage over venous blood collection in terms of convenience. Nonetheless, despite being less invasive than venous blood draws, capillary blood sampling can still cause anxiety, discomfort, and emotional distress for some patients [27], highlighting the need for further improvements in non-invasive blood testing methods.

Previously dismissed as mere biological waste, menstrual blood has recently emerged as an alternative, non-invasive sample matrix for medical diagnostics [28,29,30]. Menstrual blood is one of the components of a complex fluid known as menstrual effluent, expelled from the uterus during menstruation as a part of the menstrual cycle [31]. Besides menstrual blood, menstrual effluent also comprises vaginal secretions, cervical mucus, tissue, and cells from the reproductive tract [32], along with various microorganisms, making it a uniquely distinct biological fluid that holds promise for providing insights into the reproductive tract and associated diseases [33].

Despite remaining a relatively under-explored sample, research on menstrual effluent has increased over recent years, particularly concerning its distinct composition and unique profile. Through a comprehensive proteomic analysis, Yang et al. (2012) identified 385 proteins that are unique to menstrual blood [33]. These novel biomarkers offer opportunities for understanding and diagnosing reproductive disorders, such as endometriosis and cervical cancer [29,34,35,36,37,38].

However, besides its unique profile and complexity, menstrual blood also exhibits strong correlations with systemic blood biomarkers, including fertility-associated hormones [39,40], certain cytokines [36], hemoglobin A1c (HbA1c) [40,41], and other routine health biomarkers [28]. Zhou et al. (1989) were the first to compare biomarker concentrations in menstrual blood to those of peripheral plasma, where they analyzed various hormones [39]. The results of this preliminary study demonstrate a significant correlation between menstrual follicle-stimulating hormone (FSH) and estradiol with the corresponding peripheral levels. The significant correlation observed for FSH was further corroborated by Naseri et al. (2024) in a larger study, where menstrual luteinizing hormone (LH), Anti-Müllerian hormone (AMH), and thyroid-stimulating hormone (TSH) were also found to highly correlate with the levels in venous blood [40]. Besides hormones, such significant correlations have also been shown for other routine health biomarkers including cholesterol, creatinine, HbA1c, cholesterol, creatinine, high-density lipoprotein (HDL), low-density lipoprotein (LDL), and high-sensitivity C-reactive protein (HsCRP) [28]. This reported concordance with systemic blood underscores the premise that menstrual blood can be used as a non-invasive alternative for routine diagnostics. This has been further exemplified with the first FDA-approved HbA1c menstrual blood test, which utilizes DBS technology within a modified sanitary pad [40,41,42]. Conversely, not all biomarkers in menstrual blood correlate with those in systemic blood, as highlighted by the differences observed for glucose [28] and a large number of cytokines [36,43]. These discrepancies are thought to reflect the unique biological nature of these biomarkers within the uterine environment, owing to the distinct microbial interactions and inflammatory processes for glucose and cytokines, respectively [28,36,43]. The characterization of such biomarkers in menstrual blood, comparing healthy levels to various pathological states, could, therefore, provide valuable insights into uterine pathologies and the potential for identifying novel diagnostic approaches. Consequently, menstrual blood represents a non-invasive accessible avenue for diagnostic and therapeutic approaches, as well as regular health monitoring.

Although an important determinant of health status, to our knowledge, there are currently no published studies investigating the applicability of menstrual blood to measure vitamin levels, and therefore, no knowledge of the diagnostic potential of menstrual blood for testing for vitamin deficiencies. On the basis that various biomarkers from menstrual blood have been shown to correlate with venous blood [28,39,40], and that these are known to correlate with capillary blood biomarkers [23], we hypothesized that vitamin levels from menstrual blood would also correlate with those from capillary whole blood.

Moreover, because of the composition of menstrual effluent [33,44], we were also interested in analyzing the total hemoglobin in menstrual samples compared to capillary whole blood. We hypothesized that menstrual hemoglobin levels would be lower than capillary levels, reflecting the dilution of menstrual blood within the total menstrual fluid [45]. Previous studies have demonstrated the dilutional effect of menstrual blood, with lower hemoglobin concentrations suggesting that whole blood only accounts for a fraction (30–50%) of the menstrual fluid expelled during menstruation [46]. The dilutional effect of menstrual blood, owing to its complex composition, is one of the factors that, therefore, poses a challenge when analyzing menstrual blood biomarkers and comparing them to systemic blood levels. Menstrual FSH and estradiol levels, although strongly correlated, were found to be lower than the corresponding peripheral values, hypothesized to be a result of the dilutional effect [39]. Furthermore, the unique biological characteristics of menstrual blood that differentiate it from systemic blood, such as the presence of endometrial tissue, cervical mucous, and blood clots [33,44], are additional factors that could compromise the analyzability of menstrual biomarker levels and their comparative levels. Such components could affect the analytical aspect via interference, hinder sample handling procedures, and potentially influence the measurement of biomarkers in menstrual samples. The levels of certain cytokines [36,43], glucose [28], and prolactin [39] were all found to not correlate in menstrual blood, compared to systemic blood, with differential levels between the two matrices. These discrepancies were hypothesized to be a result of the specific biological roles of these specific biomarkers within the context of the uterine environment, for which menstrual blood is said to be representative of [47]. For example, higher prolactin levels in menstrual blood compared to peripheral blood are thought to be due to the release of prolactin from the endometrium during menstruation [39], whereas lower menstrual glucose levels are suspected to be because of the metabolic use of glucose by microorganisms within the vagina [28]. As a result, despite our aforementioned hypothesis, with no previous knowledge on vitamin levels in menstrual blood, we were intrigued to ascertain whether vitamin measurements would be possible in menstrual blood, and if possible, how these relate to those in capillary whole blood to decipher their potential diagnostic utility.

## 2. Materials and Methods

### 2.1. Inclusion Criteria as Well as Time and Organizational Procedures

**Figure 1:**
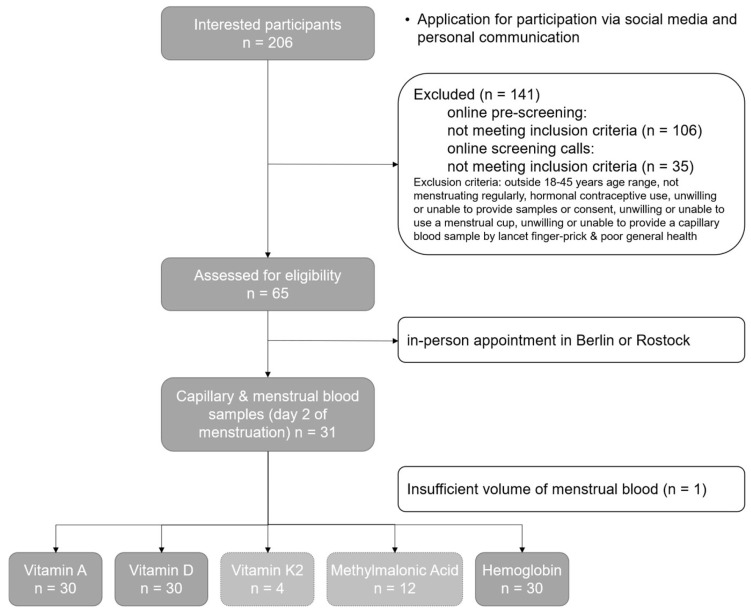
Flowchart depicting the study process from participant recruitment to sample analysis, including the number of participants at each stage and the number of menstrual and capillary blood samples collected. The bottom row of the gray boxes shows the number of paired capillary and menstrual blood samples that gave conclusive values for the respective biomarkers analyzed, with the concentrations in both sample types being above the lower limit of quantification (LLOQ).

This prospective, monocentric, cross-sectional study was carried out between May 2023 and November 2023, with 206 interested participants applying to take part. Following screening procedures, 65 participants were confirmed eligible, and 31 of them were included in the study (Figure 1).

This study was conducted to compare vitamin levels from paired menstrual blood and capillary whole blood samples. All the subjects gave their informed consent for inclusion before participation. All the participants took part in the study voluntarily with the intent of contributing to menstrual blood research and were not remunerated. The study was conducted in accordance with the Declaration of Helsinki, and the protocol was approved by the Ethics Committee of the University Medical Center Rostock (Registration number A2023-0063).

The sample size was determined using Fisher’s z-transformation (power analysis): expected correlation 0.7, correlation under null hypothesis 0.0, power 0.9, alpha 0.05, and drop-out rate 0.3. The expected correlation coefficient was based on prior studies comparing venous and capillary vitamin A [48] and vitamin D [49,50,51,52] levels in which the correlation coefficients were reported to be between 0.77 and 0.99.

The participants were healthy, regularly menstruating females of reproductive age (Table S1), recruited via social media or personal communication. The interested participants were directed to an online questionnaire to determine eligibility based on specific inclusion and exclusion criteria (Figure 1). The exclusion criteria included being outside of the 18–45 years age range, not menstruating regularly (regularly defined as menstruating every 21–35 days with 3–7 days of menstrual bleeding), hormonal contraceptive use, being unwilling or unable to provide samples or consent, unwilling or unable to use a menstrual cup, and unwilling or unable to provide a capillary blood sample by lancet finger-prick. An additional exclusion criterion was being of poor general health, which was to be determined by the study physician. Therefore, the interested participants were asked to disclose any chronic health concerns or conditions during the online questionnaire.

The eligible participants were asked to schedule an online meeting with the study physician to confirm eligibility, provide a detailed study overview, and address any questions. Additional information, such as menstruation dates and shipping addresses, was collected. The participants received an email with study details and instructions, along with a study kit via post containing an instruction sheet and a menstrual cup (The Female Company GmbH, Berlin, Germany) made of 100% medical-grade silicone.

On day 1 of menstruation, when menstrual bleeding is first noticed and a menstrual product is required, the participants were instructed to schedule an in-person appointment at one of the two study sites (Berlin or Rostock) for the following day. On day 2 of menstruation, the participants were asked to use the menstrual cup for 3–4 h before their scheduled appointment. At the study site, the participants met with the study physician to discuss study details and any concerns, and sign informed consent forms. The participants provided additional information including age, any medication or supplements taken on either day 1 or 2 of menstruation, activity level on day 2 of menstruation, pain level on a scale of 1–10 for day 2 of menstruation, emotional state (Table S1), the time of menstruation onset on day 1, and the time that the menstrual cup was inserted on day 2 (Table S2).

A lancet micro-needle device (HTL Strefa DropSafe Acti-Lance Safety Lancets, Fisher Scientific, Pittsburgh, PA, USA) was used to puncture the tip of a thumb finger and slight pressure was applied to obtain a small volume of capillary whole blood. Five drops of capillary blood were collected on a DBS card (BioSample Card, Ahlstrom Munksjö, Espoo, Finland) and left to dry for 4 h at room temperature in the dark.

To obtain the menstrual blood sample, the participants were instructed to remove the menstrual cup and pour its contents into a 50 mL falcon tube (Greiner Bio-One GmbH, Frickenhausen, Germany) in the privacy of a washroom. The time of this transfer was recorded once the participant had returned with their sample and handed it over to study personnel. Macroscopic analyses of the menstrual blood samples were carried out before they were “spotted” on the DBS cards, and photographs of all the DBS cards for both the menstrual and capillary blood samples were taken. The following visual characteristics were recorded—volume, color, and composition (blood clots and viscosity) (Table S2). The menstrual blood sample (50 µL) was pipetted onto DBS cards in triplicate. The menstrual blood DBS cards were stored in the same conditions as the capillary blood card for drying. The transfer of both the menstrual blood samples and capillary blood samples onto the DBS cards occurred within a maximum of 30 min of one another. Once dried, the three menstrual blood DBS card replicates and the capillary blood DBS card were placed in a sealable plastic bag with a desiccant and stored at −20 °C until all the samples had been collected for the study.

### 2.2. Analysis of the Samples

The DBS cards were shipped to a GMP-certified laboratory (Vitas Analytical Services, Oslo, Norway). All the cards were coded using barcodes prior to shipping to ensure the laboratory would analyze the samples in a “blinded” fashion to prevent bias.

Each DBS card was used to analyze the following biomarkers: vitamins A, D, and K2; Methylmalonic Acid (MMA); and hemoglobin, as per the laboratory’s standard operating procedures for the DBS analysis of capillary whole blood. In the case of vitamin D, the total vitamin D was measured. For vitamins A, D, and K2, DBS punches were made and diluted with water, and the analytes were extracted with 2-propanol-containing stable isotope-labeled internal standards. Extracts from the protein precipitation were analyzed using an Ultivo Triple Quadrupole Liquid chromatography-mass spectrometry (LC–MS) (Agilent Technologies, Santa Clara, CA, USA), and separation was conducted using a Kinetex^®^ 2.6 µm C18 100 Å, 100 × 4.6 mm LC column (Phenomenex, Torrance, CA, USA).

The analysis of MMA was performed using gas chromatography-coupled mass spectrometry (GC-MS). DBS spots were diluted with water and propanol, and a mix of stable isotope-labeled amino acids was added and used as internal standards. The samples were derivatized using propyl chloroformate and extracted into isooctane before analysis by GC-MS. An instrumental analysis was performed on an Agilent 6890 GC system with a split/splitless injector and a 5973N mass selective detector operated in SIM mode (Agilent Technologies, Palo Alto, CA, USA). The separation of amino acids was performed on a Zebron ZB-AAA analytical column (Phenomenex, Torrance, CA, USA), and calibration was performed using five-point calibration curves.

To analyze hemoglobin, DBS punches were made and eluted with water. The supernatant was then treated according to the manufacturer’s kit manual from the Hemoglobin Assay Kit (MAK115) by Sigma Aldrich (St. Louis, MO, USA). Absorbance was read at 400 nm using a Spark 10M microplate reader (Tecan Life Sciences, Männedorf, Switzerland).

Values for vitamin A, vitamin D, and hemoglobin were obtained for all the menstrual blood samples and the corresponding capillary blood samples (Figure 1). However, for both the menstrual and capillary blood samples, a large number did not provide exact values for vitamin K2 nor MMA due to the concentrations being less than the lower limit of quantification (LLOQ) (Figure 1). Therefore, no further analyses were conducted for these two biomarkers.

### 2.3. Statistical Analysis

The results for each of the three menstrual blood DBS cards, for each sample, were calculated as a mean, and compared to the value of the corresponding capillary blood DBS card for statistical analyses. Data wrangling was performed using Python 3.10.8 with the pandas 1.5.2 and NumPy 1.23.2 libraries. For data visualization, the matplotlib 3.6.2 and seaborn 0.12.2 libraries were used. A two-sided, two-sample (paired) *t*-test with no equal variance was carried out to determine the statistical significance of the difference between the two sample types. Confidence intervals were calculated to assess the variation around the mean menstrual blood value and the capillary blood value. A regression analysis was performed between the menstrual and capillary paired samples in order to deduce whether the capillary blood values can be predicted using the mean menstrual blood results. Pearson’s correlation coefficient (SciPy 1.10.0 library) was used to determine the extent of the linear relationship between the capillary blood and menstrual blood values, where *r* values closer to 1 or −1 signify a strong positive and negative correlation, respectively, with those closer to 0 indicating no correlation. For statistical significance, a value of *p* < 0.05 was considered significant. A Bland–Altman analysis (Pingouin 0.5.3 library) was performed to visualize the differences between the menstrual blood and capillary blood results to help identify whether the two sample types sufficiently agree to be used interchangeably with one another.

## 3. Results

Healthy, reproductive-aged individuals were recruited to take part in this study, with the interested participants younger than 18 years old and older than 45 years old excluded (Figure 1). The average age of the included participants (n = 31) was 30.4 ± 4.8 years (mean ± Standard deviation (SD)), with an age range of 20–41 years old (Table S1). The menstrual and capillary blood samples were collected from each of the participants (n = 31) on day 2 of menstruation of the same menstrual cycle (Figure 1). The menstrual blood samples were collected using a menstrual cup with an average collection time of 4.9 ± 2.6 h (mean ± SD) (Table S2). All the menstrual blood samples collected were of sufficient volume, except for one, which was less than 750 µL and thus not sufficient to transfer onto the triplicate DBS cards (Figure 1). Therefore, the menstrual and capillary blood samples of this participant were excluded from the subsequent steps. However, the majority (90.7%, n = 29) of the included menstrual blood samples (n = 30) were above 1 mL in volume, with over half (54.8%, n = 17) being 5 mL or above (Table S2).

All the menstrual and capillary blood samples of adequate volume (n = 30) provided values for vitamin A, vitamin D, and hemoglobin levels following analysis (Figure 1), allowing for comparison analyses on the paired samples for these parameters. The results revealed statistically significant differences (*p* < 0.001) in the mean levels of these analytes between the two sample types (Table 1). The confidence intervals between the mean differences for the three biomarkers show the range of mean differences at the 95% confidence interval. This was further corroborated following a paired *t*-test analysis of the data, which showed that the values from the menstrual blood samples are statistically significantly different from those from the capillary blood samples. In all the instances, the mean menstrual blood values were lower than the corresponding capillary blood values (Table 1).

Despite the statistically significant differences observed between the values of the capillary blood and menstrual blood for vitamin A and vitamin D, it could be that they are still related to one another. As a result, correlation analyses were performed to see if this was the case. From this, it could be confirmed that for both vitamins A and D, there is a relationship between the menstrual and capillary values (Table 2).

**Figure 2:**
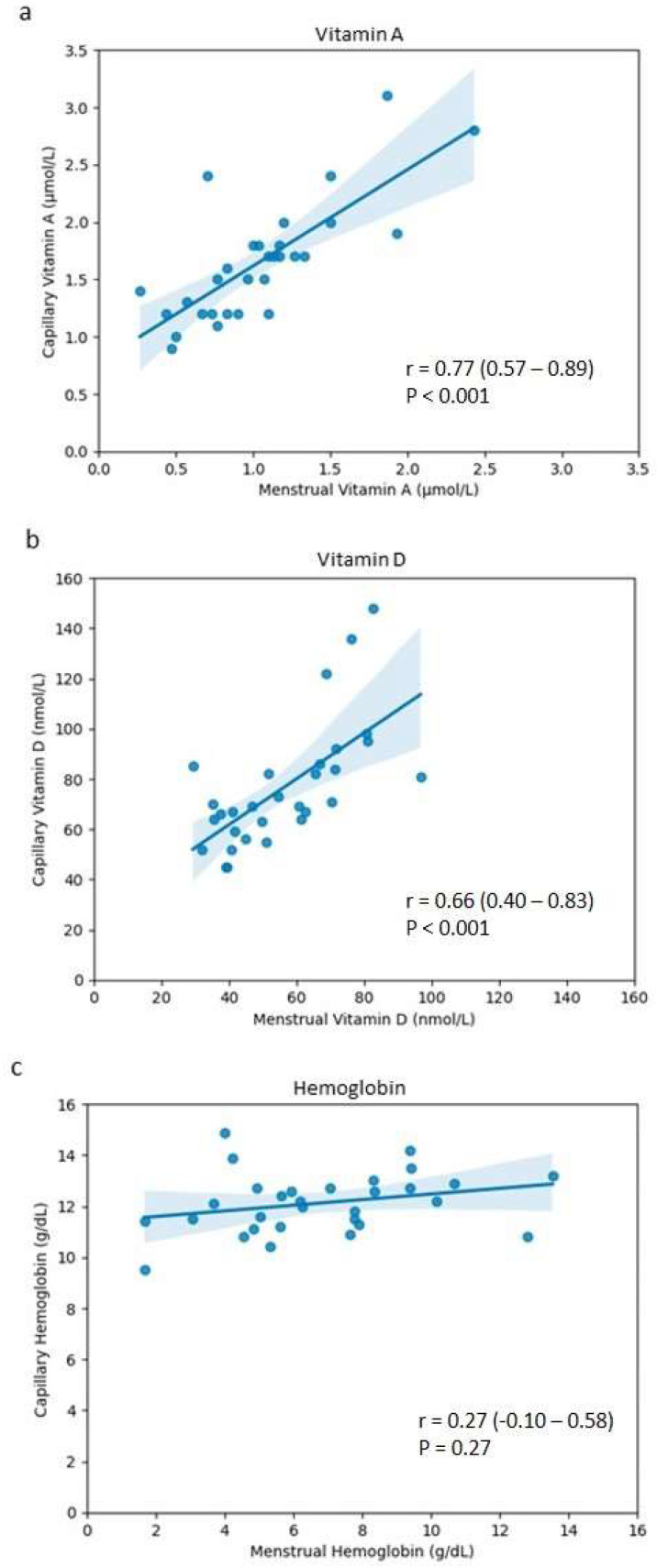
Pearson’s correlation coefficient plots between the menstrual blood and capillary blood for (**a**) vitamin A, (**b**) vitamin D, and (**c**) hemoglobin. The blue line indicates the linear regression model fit of the data, while the blue shaded area indicates a 95% confidence interval for that regression.

For vitamin A, the menstrual and capillary blood values are strongly correlated to one another (Table 2, Figure 2a). This was also the case for vitamin D, albeit with a more moderate, but still significantly positive, correlation between the values (Table 2, Figure 2b). However, no correlation is apparent between the values for hemoglobin (Table 2, Figure 2c).

**Figure 3:**
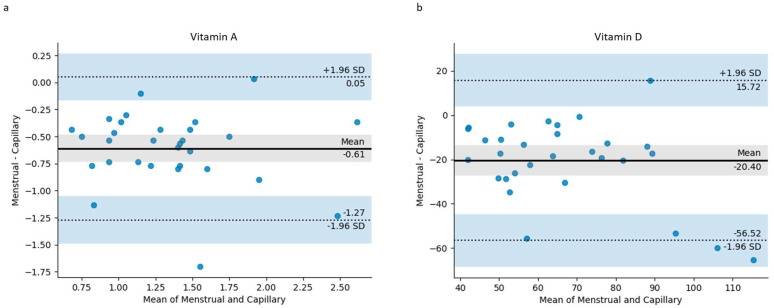
Bland–Altman plots of differences versus the means of the menstrual and capillary blood values measured for (**a**) vitamin A and (**b**) vitamin D. The mean difference is illustrated by the solid black line (inside the gray area) parallel to the *x*-axis. The limits of agreement are indicated by the dashed lines parallel to the *x*-axis at −1.96 SD and +1.96 SD inside the two blue-shaded regions. The gray-shaded area represents the 95% confidence interval limits for the mean difference, and the blue-shaded region shows the 95% confidence interval for the agreement limits.

Bland–Altman plots were used to visualize the differences in measurements between the two matrices, menstrual blood and capillary blood, for vitamins A and D (Figure 3). From these, when comparing the menstrual and capillary blood measurements of vitamin A (Figure 3a), the mean difference (bias) was found to be −0.61, indicating that the menstrual measurements were, on average, lower than the capillary measurements. The limits of agreement, calculated as the mean difference plus and minus 1.96 times the standard deviation, were 0.05 and −1.27. Most of the data points observed in the plot fell within these limits of agreement, demonstrating a strong agreement between the menstrual and capillary blood for vitamin A.

Similarly, the Bland–Altman plot for vitamin D (Figure 3b) shows a mean difference of −20.40, showing that like vitamin A, the menstrual blood values are typically lower than the paired capillary blood sample. With the exception of two outliers that fall outside the limits of agreement (+15.72 and −56.52), there appears to be a strong degree of agreement between the menstrual and capillary blood for measuring vitamin D levels.

## 4. Discussion

The routine measurement of clinically relevant vitamin levels is essential for maintaining overall health due to their fundamental roles in numerous physiological functions within the body [1,20]. However, the inconvenience and emotional distress associated with the current blood testing methods may deter individuals from regular testing [27]. Menstrual blood has been proven as a potential non-invasive and more convenient alternative for routine diagnostics, which could help address these barriers [28,35,40,41]. Recent studies have revealed the extent of this potential, with menstrual blood values for various routine biomarkers, including hormones and triglycerides, showing strong correlations with systemic blood [28,40,53]. The routine testing of HbA1c, one of the gold-standard biomarkers for diagnosing and monitoring prediabetes and diabetes [54], is now possible using menstrual blood via a specifically adapted sanitary pad that incorporates DBS technology [40,42]. As the first approved menstrual blood health test, this HbA1c test reflects the average levels over the past three months, enabling the monitoring of blood sugar levels for those with diabetes as a more accessible alternative to invasive venous blood draws [40,41]. Despite increasing interest in the roles of various vitamins and menstrual health [8,55,56,57], and the clinical relevance of vitamins such as vitamin D [58], the possibility of using menstrual blood to analyze vitamin levels was unclear until now. In this study, using menstrual blood collected in menstrual cups and DBS technology, we assessed the feasibility of testing vitamin levels in menstrual blood, consequently revealing its plausibility for both vitamins A and D.

Vitamins A and D are fat-soluble compounds integral to numerous cellular processes and the prevention of diseases [59,60,61,62,63]. Vitamin A plays key roles in immunity, and cellular differentiation and growth, with insufficiencies associated with night blindness and a weakened immune system, and supplementation linked to a lowered incidence of ovarian cancer [64,65]. Vitamin D, on the other hand, is indispensable for proper organ function, and bone and cardiovascular health [3,66], with deficiencies leading to osteomalacia [67,68] and linked to autoimmune diseases and certain cancers [69,70,71]. Recent research has also uncovered an important role of vitamin D in reproductive health [72], with associations demonstrated for infertility [73], polycystic ovary syndrome (PCOS) [57,73,74], and endometriosis [75]. For instance, lower vitamin D serum levels have been found to be associated with increased risk of PCOS [76] and differing levels have been observed between individuals with and without endometriosis [77,78,79,80,81,82]. Thus, the regular monitoring of both vitamin A and D, enabling timely interventions through diet and supplementation, is important for ensuring good health, particularly in reproductive-aged women.

For this study, 206 interested participants volunteered to take part, showing the willingness of menstruating individuals to donate menstrual blood for such purposes. A recent study in France also showed such willingness, where 78% of the menstruating women reported that they would donate menstrual blood for research regarding endometriosis [83]. In the United States of America (USA) [28], however, one study stated that some women declined to participate due to having to use a menstrual cup as the collection method, leading the authors to declare menstrual cup use as a limitation. In our study, 66% (n = 132) of the asked applicants (n = 199) stated that they regularly used a menstrual cup, and 89% (n = 59) of the applicants who had never used a menstrual cup before (n = 66) claimed that they would be willing to in order to take part in the study. Therefore, menstrual cups were not seen as a limiting factor in our participant recruitment process, which could be a potential reflection of the geographical differences in attitudes towards menstrual cups [84], the global increase in menstrual cup use [85], or the result of bias via the recruitment of individuals through personal communication.

The average duration of menstrual cup wear was 4.9 h, during which sufficient volumes of menstrual blood were collected for all the participants except one (Figure 1) given the lower volumes required for DBS technology as opposed to alternative methods [86]. Additionally, menstrual cup collection in conjunction with DBS technology seemed sufficient for the analysis of vitamins A and D, given that menstrual blood samples of adequate volume were analyzable and values were obtained (n = 30) (Figure 1).

However, for vitamin K2 and MMA, the data were insufficient, particularly from the capillary blood samples. For MMA, we received results from only 43% (n = 30) of the capillary DBS cards compared to 66% from the triplicate menstrual blood cards (n = 90). Similarly, for vitamin K2, we obtained results from 20% (n = 30) of the capillary blood samples compared to 54% (n = 90) from the menstrual DBS cards. This discrepancy can be attributed to the low concentrations of these biomarkers in systemic blood for a healthy population [87,88]. In the absence of supplementation, vitamin K2 blood concentrations are typically low, with one study reporting serum levels of 1.553 ± 0.226 ng/mL in premenopausal women using high-performance LC-MS/MS [89]. Due to an LLOQ of 2 ng/mL for the LC-MS DBS methodology employed in this current study, a large proportion of the samples had vitamin K2 levels lower than the detection limit, whereby exact values could not be provided. The same applies to MMA, where the normal distribution is 0.07–0.27 µmol/L [90], the LLOQ for DBS LC-MS is 0.05 µmol/L, and the clinical cut-off is 0.4 µmol/L, meaning that for the majority of the participants without elevated MMA levels, an exact value could not be obtained and provided. Therefore, the typically low concentrations of these biomarkers for a healthy population resulted in a significant number of values below the LLOQ for both the capillary and menstrual samples, thus reducing the sample sizes to a point where meaningful analysis was not possible. Consequently, we excluded vitamin K2 and MMA from the final results due to the insufficient data obtained from the capillary DBS cards. As a limitation in our study design, future studies should take this factor into account and analyze biomarkers in menstrual blood from individuals with known levels above the LLOQs of the chosen method.

For vitamins A and D, the analysis of the values obtained illustrated that the menstrual blood levels show statistically significant correlations with the capillary blood values (Table 2; Figure 2). The Bland–Altman analysis also shows an agreement between measuring these biomarkers in both sample types (Figure 3), with the overwhelming majority of data points for both vitamin A and vitamin D being within the limits of agreement. This observation is an indication that menstrual blood can be used as an alternative to capillary blood for these two analytes. To our knowledge, this is the first study to compare vitamin A and D levels between menstrual and capillary blood. Previous studies have demonstrated the concordance of menstrual blood to venous blood for several biomarkers including FSH [39,40], estradiol [39], LH [40], AMH [40], and TSH [40], as well as for a number of routine diagnostic biomarkers: HbA1c, cholesterol, creatinine, HDL, LDL, and HsCRP [28,40,41]. For all of the aforementioned biomarkers, similarly strong correlations were observed between menstrual blood and venous blood, as was observed in this study for the menstrual and capillary blood.

Although correlations for vitamins A and D were observed, our findings indicate no correlation between the hemoglobin levels in menstrual blood and those in capillary blood (Figure 3). This discrepancy may be due to the composition of the menstrual blood itself, along with the differing biological characteristics between these specific biomarkers. Hemoglobin is an iron-containing protein confined within red blood cells [91]. As a complex biological fluid, blood only represents one of the distinct fractions within menstrual blood [92]. The menstrual fluid excreted during menstruation, known as menstrual effluent, is a mixture of blood, vaginal secretions, cervical mucus, and endometrial tissue [32,44]. Consequently, the blood component of menstrual blood may be diluted by the non-blood components, resulting in a lower proportion of red blood cells and thus reduced hemoglobin concentrations compared to capillary whole blood. The lower levels of hemoglobin observed in the menstrual blood (Table 1), compared to the capillary blood, support this notion, as do the lower levels observed for other biomarkers analyzed in previous studies [28,39]. The hemoglobin levels in the menstrual blood were significantly lower than those in the capillary blood (Table 1), consistent with previous findings that both hemoglobin and hematocrit are lower in menstrual blood compared to peripheral blood [39,92,93]. These findings suggest a dilution effect in menstrual blood regarding its blood component. However, since hemoglobin in menstrual blood does not correlate with capillary blood (Table 2, Figure 2), this dilution does not appear to be uniform, with possible variation between menstrual samples. Indeed, a Bland–Altman plot for hemoglobin shows a distinct linear trend, suggesting that non-uniform sample dilution could have occurred (Figure S1). One study analyzing menstrual effluent from 28 women found that the proportion of the blood component varies greatly between individuals, ranging from 1.6% to 81.7%, indicating significant individual differences in the composition of menstrual effluent [46]. Therefore, assuming hemoglobin is a biomarker present only within the blood component of menstrual effluent, this variation likely accounts for the lack of correlation between the menstrual blood and capillary blood observed in the present study.

By comparison, fat-soluble vitamins such as vitamins A and D, as well as water-soluble vitamins, are systemic, meaning that they are not cell-bound [9], and thus may be present both in the blood and non-blood components of menstrual effluent. This could explain the significant correlations observed for vitamins A and D in the menstrual blood to the levels in the capillary blood (Table 2, Figure 2), suggesting that the concentrations of these biomarkers are not subject to the non-uniform sample dilution effect affecting the blood proportion of the menstrual samples, as with hemoglobin.

Nevertheless, despite the significant correlations, our results also demonstrate that the menstrual blood values are not identical to those in the capillary blood for vitamins A and D, with statistically significant differences between the values obtained (Table 1). When analyzing the mean differences from the menstrual and capillary blood values (Table 1), and plotting these with the Bland–Altman analysis (Figure 3), it is clear that for both vitamin A and vitamin D, the values are lower in the menstrual blood than in the capillary blood, although still correlated (Table 2; Figure 2). Similarly, previous studies also showed this to be the case for other biomarkers analyzed in menstrual blood when compared to venous blood, such as for FSH, LH, creatinine, and triglycerides [28,39]. Therefore, taken together, the biomarker analyses and subsequent statistical analyses suggest that vitamin A and D levels are different when measured in menstrual blood compared to those of the corresponding capillary samples. Nevertheless, it is plausible to assume that the values for both of these vitamins from the menstrual blood can be used to predict the capillary values, given the significant correlations observed for both, and based on most of the data points being within the limits of agreement, as shown in the Bland–Altman plots (Figure 3).

As correlations were observed for both vitamins A and D (Table 2; Figure 2), unlike for hemoglobin, sample dilution is unlikely the sole cause for the significantly lower levels in the menstrual blood compared to the capillary blood (Table 1). Yet, in another, larger study, whereby menstrual blood was collected using a modified sanitary pad, as opposed to a menstrual cup, FSH levels in menstrual blood were not seen to be statistically different from those of the corresponding venous blood [40]. The previously reported differences between menstrual and venous blood FSH values could have, therefore, been an effect of the small sample size or may have been attributed to the menstrual cup use as the menstrual blood collection method, despite the significant correlations [28,39]. For glucose, however, statistically significant differences were observed with no correlation between the menstrual and systemic values, possibly due to the metabolism of glucose by microorganisms in menstrual blood [28].

It is unclear whether the observed discrepancies in the present study between vitamins A and D in the menstrual blood compared with the capillary blood reflect biological differences in these vitamin levels between the two matrices, or whether they are a result of sample collection or processing methods. Both samples, once transferred to the DBS cards, were subjected to the same treatment in terms of storage, shipping, and laboratory testing to limit such effects, so although possible, it is unlikely that the differences accrued as a result of these procedures.

However, the menstrual blood samples were collected in menstrual cups for 4.9 h on average before being transferred to the DBS cards (Table S2). As the majority of the collected menstrual blood samples were dark red (Table S2), possibly due to oxidation [94], it is likely that sample quality prior to analysis was affected due to degradation and/or hemolysis. This degradation of the menstrual blood samples could have contributed to the differences observed by impacting the stability of the analytes or the performance of the analytical techniques. Both temperature and time have been reported to affect the stability of vitamins A and D [95,96]. Therefore, the effect of these factors should be explored further during menstrual blood collection. With no current knowledge of the stability of vitamins A and D in menstrual blood over time or at different temperatures, more thorough analyses investigating such variables would need to be carried out to determine whether these differences between the capillary and menstrual blood levels are due to analyte stability in the menstrual blood, or possible alternative factors.

For instance, the lower levels of vitamins A and D in the menstrual blood compared to the capillary blood could be a reflection of the inherent biological characteristics of these analytes in the menstrual blood, and not a result of sample and analyte stability, or sample dilution. Both vitamins are known to have anti-inflammatory effects [97,98,99,100], and are utilized in processes such as tissue repair and inflammation response, which are heightened during menstruation [101,102]. Vitamin D is believed to have a direct menstrual cycle-related role and is involved in the modifications of the endometrium throughout the menstrual cycle [103,104]. Additionally, a vitamin D receptor has also been found within the human endometrial tissue [105], and enzymes involved in the metabolism of vitamin D are also expressed in this tissue [106]. Thus, the metabolism of vitamin D during menstruation could account for the lower levels observed in the menstrual blood. Given the known anti-inflammatory roles of vitamin A and its associations with gynecological conditions [6], the same could be true for vitamin A with local metabolism within the uterus leading to reduced levels in the menstrual blood. More research is needed to investigate the mechanisms of actions of vitamins A and D, within the context of the menstrual cycle and reproductive health, to ascertain whether this is a plausible, contributory factor as to why there are lower, yet still correlated levels, in menstrual blood than in capillary blood. Moreover, as total vitamin D was measured in the present study, further research involving the analysis of free vitamin D levels in menstrual blood could prove beneficial in gaining clarity surrounding the biological action of vitamin D in relation to menstrual health [107]. This would be particularly interesting given the presence of vitamin D-binding protein (VDBP) in cervicovaginal fluid [108], which is one of the non-blood components within menstrual blood, and there are known associations of VDBP with reproductive health and disorders [109].

As a proof-of-concept study, we have shown promising initial results that illustrate the potential feasibility of measuring vitamins in menstrual blood and thus the possibility of a menstrual blood vitamin test, particularly for vitamins A and D. However, this study’s limitations, including the small sample size, limit the representativeness of the results and highlight the need for further research with larger populations to validate these findings. Such studies should also analyze sample and analyte stability, evaluate clinical and analytical validity, and establish clinical reference ranges for vitamins in menstrual blood. As there is evidence to suggest that vitamin levels are influenced by factors like age [110], future studies should also consider such factors and their potential impact on menstrual blood vitamin levels. Given the small sample size of the present study, conclusions could not be drawn from the data on the impact of age. With larger, more robust studies, a vitamin menstrual blood test of clinical accuracy may be an eventuality, which is ultimately comparable to the current alternative methods. Importantly, it is worth noting that the results of this initial pilot study are representative and limited to the use of menstrual cups being used as the menstrual sample collection method, and analysis via DBS technology. This method and, therefore, the results obtained may not be indicative of other collection methods or analysis methods, which should be a limitation to be considered when interpreting the data in terms of their broader applicability.

## 5. Conclusions

As a pilot study, the findings that vitamin A and D levels in menstrual blood correlate with those in capillary blood offer insights into the possibility of a non-invasive, convenient alternative to testing these vitamins, as well as being yet another example of the potential of menstrual blood for routine diagnostics. The analyses of menstrual blood, like in the present study, not only provide evidence for the potential of menstrual blood as a non-invasive diagnostic specimen in healthcare, but also contribute to reducing the negative stigma associated with menstrual blood. Consequently, such analyses support promoting the use of menstrual blood for further research, ultimately aiding with efforts to improve healthcare outcomes for women.

## Figures and Tables

**Table 1:** Comparison of values from the menstrual blood and capillary blood paired samples for vitamin A, vitamin D, and hemoglobin (n = 30). A value of *p* < 0.05 was considered significant.

Biomarker (Units)	Capillary Blood Mean ± SD	Menstrual Blood Mean ± SD	Paired *t*-Test (*p* < 0.05)	Mean Difference	95% Confidence Interval of the Mean Difference
Vitamin A (µmol/L)	1.65 ± 0.52	1.04 ± 0.47	<0.001	0.61	0.36–0.86
Vitamin D (nmol/L)	76.60 ± 24.51	56.20 ± 17.76	<0.001	20.40	9.57–31.23
Hemoglobin (g/dL)	12.12 ± 1.12	6.76 ± 2.93	<0.001	5.36	4.23–6.49

**Table 2:** Pearson’s correlation coefficients between the menstrual and capillary values for vitamin A, vitamin D, and hemoglobin (n = 30). A value of *p* < 0.05 was considered significant.

Biomarker	Pearson’s Correlation Coefficient (*r*)	*p*-Value	95% Confidence Interval
Vitamin A	0.77	<0.001	0.57–0.89
Vitamin D	0.66	<0.001	0.40–0.83
Hemoglobin	0.27	0.14	−0.10–0.58

## Data Availability

The data presented in this study are available upon request from the corresponding author. The data are not publicly available but can be obtained from the Department of Clinical Research at the Orthopedic Department of the University Medicine Rostock if required.

## Supplementary Materials

The following supporting information can be downloaded at https://www.mdpi.com/article/10.3390/jcm13237212/s1, Table S1: Participant self-reported demographic and clinical information; Table S2: Menstrual blood sample characteristics; Figure S1: Bland–Altman plot of differences versus the means of menstrual and capillary blood values measured for hemoglobin.
