# Supplementary material for: Menstrual Blood as a Non-Invasive Alternative for Monitoring Vitamin Levels
Source: J Clin Med. 2024 Nov 27;13(23):7212. doi: 10.3390/jcm13237212 (PMC11642367; doi:10.3390/jcm13237212)
Supplement: Supplementary file 1 [file jcm-13-07212-s001.zip › jcm-3319968-supplementary.pdf]

Supplementary Materials

Table S1. Participant self-reported demographic and clinical information (n = 31). Day 1 refers to the day when menstruation starts and day 2 is the day of sample collection. Dashes (-) indicate that no data were collected regarding the characteristic for that day.

**Mean Age ± Standard deviation (years)**	**30.4 ± 4.8**	
**Age Range (years)**	**20–41**	
**Self-Reported Participant Data**	**Day of Menstruation**	
	**Day 1**	**Day 2**
**Medication, n (%)**		
Yes	14 (45.2)	7 (22.6)
Pain medication	8 (25.8)	3 (9.7)
Other	6 (19.3)	4 (12.9)
No	17 (54.8)	24 (77.4)
**Supplements, n (%)**		
Yes	13 (41.9)	11 (35.5)
Vitamin A	3 (9.7)	1 (3.2)
Vitamin D	6 (19.3)	4 (12.9)
No	18 (58.1)	20 (64.5)
**Activity level, n (%)**		
Very active	-	0
Not very active	-	19 (61.3)
Short and intensive	-	3 (9.7)
Moderately active	-	9 (29.0)
Not active	-	0
**Menstrual-related pain, n (%)**		
Yes	-	17 (54.8)
Average pain level = 3/10 (moderate pain)	-	3 (9.7)
Highest pain level = 7/10 (very severe pain)	-	1 (3.2)
No	-	14 (45.2)
**Frequently reported emotional states, n (%)**		
Stressed	-	14 (45.2)
At peace	-	11 (35.5)
Motivated	-	10 (32.3)
Okay	-	10 (32.3)
Mood swings	-	9 (29.0)
Relaxed	-	9 (29.0)
Quiet	-	9 (29.0)

Table S2. Menstrual blood sample characteristics (n = 30). All the characteristics were determined from the liquid menstrual blood sample, before being transferred to the dried blood spot (DBS) cards.

**Mean duration of menstrual cup usage ± Standard deviation (hours)**	**4.9 ± 2.6**	
**Characteristic of menstrual blood sample**	**Classification**	**n (%) of samples**
**Volume (ml)**	< 1	1 (3.3)
	≥ 1 and < 5	12 (40)
	≥ 5 and < 10	14 (46.7)
	≥ 10	3 (10)
**Viscous**	Yes	15 (50)
	No	15 (50)
Color	Red	1 (3.3)
	Dark Red	23 (76.7)
	Brown	3 (10)
**Visible Clots**	Yes	14 (46.7)
	No	16 (53.3)

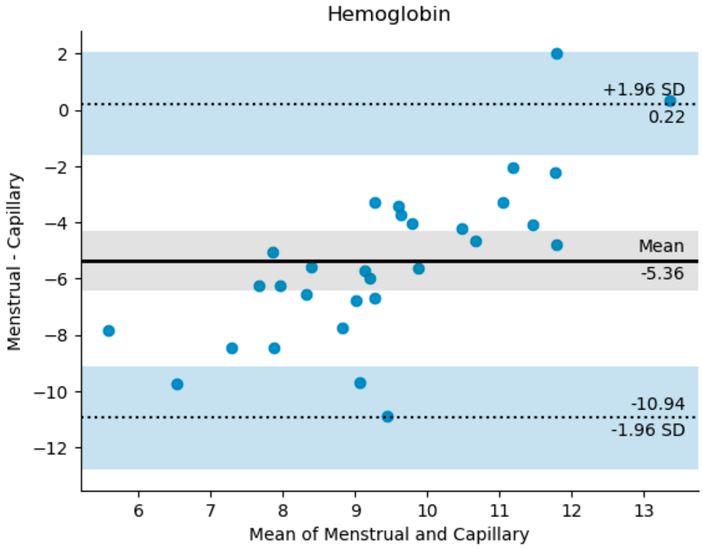

Figure S1. Bland–Altman plot of differences versus the means of menstrual and capillary blood values measured for hemoglobin. The mean difference is shown by the solid black line (inside the gray area) parallel to the *x*-axis. The limits of agreement are illustrated by the dashed lines parallel to the *x*-axis at -1.96 SD and +1.96 SD, inside the two blue-shaded regions. The gray-shaded area indicates the 95% confidence interval limits for the mean difference, and the blue-shaded region shows the 95% confidence interval for the agreement limits.
